# A Case of Spontaneous Coronary Artery Dissection

**DOI:** 10.7759/cureus.53174

**Published:** 2024-01-29

**Authors:** Connor Rougelot, Micah Pippin

**Affiliations:** 1 Family Medicine, Louisiana State University Health Sciences Center, Alexandria, USA

**Keywords:** acute coronary syndrome (acs), st elevations, coronary artery dissection (cad), atypical spontaneous coronary artery dissection, spontaneous coronary dissection

## Abstract

Spontaneous coronary artery dissection (SCAD) is the development of an intramural hematoma that causes a false lumen to form, leading to occlusion and ischemia. It is a rare but separate pathologic cause of acute coronary syndrome, more commonly occurring in females and often associated with underlying vascular conditions. Definitive diagnosis requires invasive coronary angiography. Management is similar to that of myocardial infarction caused by atherosclerosis; however, the majority of SCADs are managed conservatively, as stenting often leads to worse outcomes. Diagnostic and management strategies are primarily based on consensus, with minimal randomized control trials or prospective analyses available to guide patient care.

## Introduction

Spontaneous coronary artery dissection (SCAD) is the acute development of an intramural hematoma in a coronary artery not caused by atherosclerotic plaque rupture, intramural thrombus, trauma, or manipulation [1-6]. Clinical presentation and symptoms are similar to atherosclerotic coronary disease; however, they typically occur in younger patients and have a higher prevalence in females (up to 35% of myocardial infarctions in women less than 50 years of age) [3]. The true prevalence of SCAD remains uncertain as it is considered an underdiagnosed condition; however, data suggest SCAD accounts for approximately 1% to 4% of cases of acute coronary syndrome (ACS) [3].

SCAD has also been reported as the most common cause of pregnancy-associated myocardial infarction at 43% [3]. Some data suggest higher rates of SCAD in white populations; however, this appears to be more so from study bias and not confirmed, with cases of SCAD reported in black, Hispanic, and other racial populations. Traditional risk factors for atherosclerotic heart disease, such as hypertension, hyperlipidemia, diabetes, and smoking, are important to consider. However, these comorbidities may be lower in patients presenting with SCAD.

Appropriate diagnosis is crucial in the management of SCAD as the treatment slightly differs from myocardial infarction caused by plaque or thrombus. A diagnosis of SCAD is confirmed by coronary angiography, and it is often managed more conservatively, as percutaneous coronary intervention (PCI) and thrombolysis have been associated with poor outcomes.

In this case, we discuss an atypical presentation of SCAD in a patient not fitting the *typical* age, gender, and racial categories reported in the available literature.

## Case presentation

A 75-year-old male with a past medical history of hypertension, chronic obstructive pulmonary disease, and prostate cancer in remission for three years presented to the emergency department via emergency medical services (EMS) with a chief complaint of weakness and shortness of breath. The patient reported symptoms for the past week that had progressively worsened. Symptoms included shortness of breath, dry cough, diarrhea, dysuria, and subjective fever. A review of the systems was negative for chills, body aches, dizziness, lightheadedness, and chest pain. Progressive weakness resulted in a near fall, prompting the patient’s wife to call EMS.

The patient lived independently with his wife. He was ambulatory with the assistance of a cane. He reported occasional alcohol use and denied recreational drug use. He was a former smoker with a 40-pack-year history; however, he had quit two years before admission.

**Figure 1:**
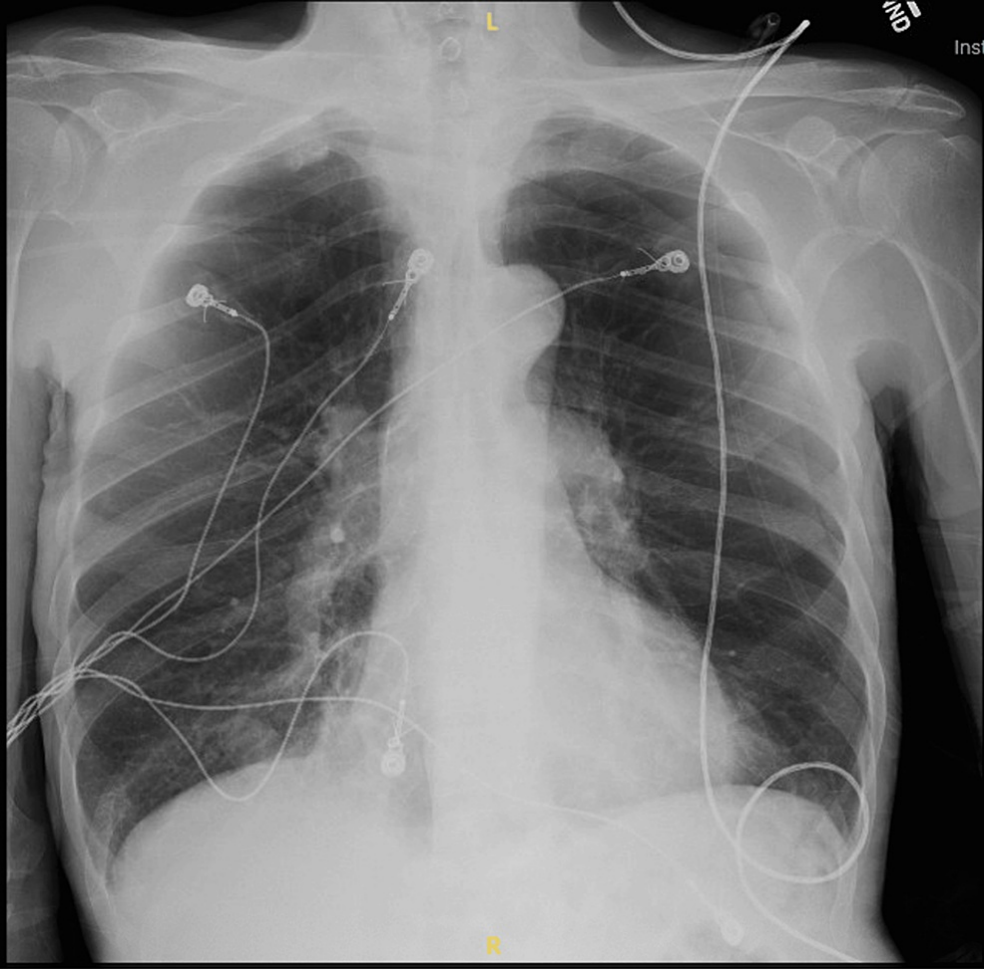
Chest radiography with no acute process identified.

Initial vital signs were significant for tachycardia in the 110 to 120 beats per minute range. The patient required 4 L of supplemental oxygen to maintain oxygen saturation greater than 90%. The physical examination was mostly benign. He was a thin elderly male, alert and oriented, in no acute distress, tachycardic in regular rhythm, with decreased breath sounds but no respiratory distress. No jugular venous distention or peripheral edema was noted. Diagnostic evaluation was significant for leukocytosis of 19.7 k/mm^3^ (normal = 5.0-10.0 k/mm3) and mild normocytic anemia with a hemoglobin of 10.7 g/dL (normal = 12.0-16.0 g/dL). The metabolic panel was unremarkable. Procalcitonin was elevated at 0.54 ng/mL (normal = <0.1 ng/mL), and lactic acid at 3.3 mmol/L (normal = 0.7-2.5 mmol/L). D-dimer was elevated at 1.63 mg/L (average = 0-0.50 mg/L). Laboratory values are summarized in Table 1. Chest radiography demonstrated no localized consolidation or pulmonary effusion (Figure 1). Subsequent computed tomography angiography (CTA) of the chest was negative for pulmonary embolism; however, there were findings of chronic obstructive pulmonary disease. Initial troponin was negative at <0.01 ng/mL (normal = 0.0-0.1 ng/mL). The patient’s urinalysis was consistent with a urinary tract infection. The patient also tested positive for COVID-19 and influenza A.

**Table 1:** Relevant laboratory values.

	Laboratory values	Reference ranges
Leukocytes	19.7 k/mm^3^	5.0–10.0 k/mm^3^
Hemoglobin	10.7 g/dL	12.0–16.0 g/dL
Procalcitonin	0.54 ng/mL	<0.1 ng/mL
Lactic acid	3.3 mmol/L	0.7–2.5 mmol/L
D-dimer	1.63 mg/L	0–0.50 mg/L
Troponin	<0.01 ng/mL	0.0–0.1 ng/mL

**Figure 2:**
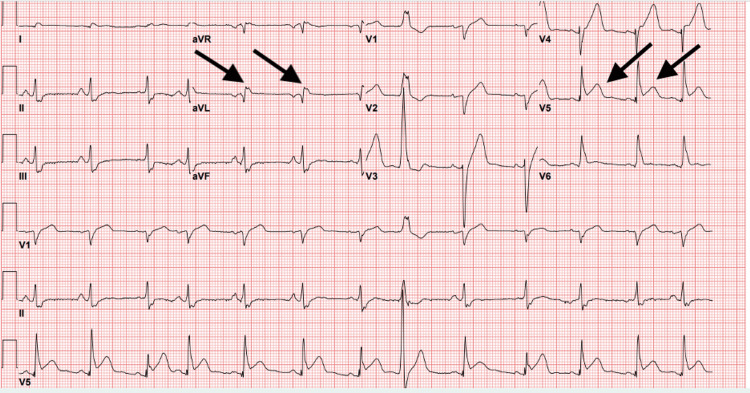
Electrocardiogram with ST-segment elevation in the lateral leads.

The patient met the sepsis criteria and was admitted for antibiotics and intravenous fluids, as well as respiratory support. Initial echocardiography showed an ejection fraction of 40% with septal motion abnormalities consistent with a left bundle branch block. On hospital day four, the patient developed an acute change. Telemetry monitoring revealed a supraventricular tachycardia rhythm with a rate of 180 beats per minute; however, the patient had no new symptoms, denying chest pain or increased shortness of breath. The dysrhythmia converted with vagal maneuvers and beta-blockade. Subsequent electrocardiogram (ECG) showed lateral ST-segment elevation, which was not present on prior ECG (Figure 2). Repeat troponin was again negative at <0.01 ng/mL.

The patient was taken to the catheterization lab due to persistent ST-segment elevation on ECG. Coronary angiography showed SCAD of the obtuse marginal branch of the left circumflex artery, as well as severely decreased left ventricular ejection fraction of 20%. As the patient was clinically stable, the decision was made to manage the coronary dissection with conservative medical management. Due to severely decreased left ventricular ejection fraction, an intra-aortic balloon pump was implanted, and the patient was transferred to the intensive care unit.

The patient continued to improve, and the intra-aortic balloon pump was removed the following day. He was treated medically with aspirin and atorvastatin. Once his blood pressure improved, the patient was started on Entresto and fitted for a Life Vest before discharge on hospital day 11.

## Discussion

SCAD is an important cause of ACS with a pathology that differs from the more common presentations of ACS. This atypical presentation is more common in women, accounts for up to 35% of ACS in women less than 50 years old, and is the most common cause of pregnancy-related myocardial infarction [2,3]. SCAD results from the formation of an intramural hematoma, with or without damage to the endothelium, that causes the development of a false lumen, leading to occlusion and ischemia [1,2]. Underlying vascular conditions are often associated with a diagnosis of SCAD, most commonly fibromuscular dysplasia (FMD). Therefore, some recommendations include screening for FMD or other vascular abnormalities at the time of SCAD diagnosis. Other inherited vascular and connective tissue diseases such as Ehler-Danlos, Marfan syndrome, and Loeys-Dietz syndrome have also been associated with SCAD; however, it does not appear to be familial [4]. SCAD can occur in any coronary artery; however, it more commonly occurs in the left anterior descending artery and usually involves mid to distal segments [3].

Clinical presentation is similar to that of atherosclerotic ACS, with patients complaining of chest pain, shortness of breath, diaphoresis, syncope, and dizziness. Cardiac biomarkers are usually elevated with ECG tracings similar to ST-segment elevation myocardial infarction or non-ST-segment elevation myocardial infarction. SCAD can also cause ventricular dysrhythmias, cardiogenic shock, and even cardiac arrest. The diagnosis is based on clinical suspicion but confirmed with coronary angiography. Multiple types of SCAD have been described using the Yip-Saw classification system (Table 2). The most common Type 2 appearance is characterized by a long smooth narrowing, often tapering distally, either with distal reconstitution of a normal vessel or extending into distal branches [4]. The less common Type 1 presentation shows an identifiable false lumen with a linear filling defect or dissection flap. The contrast will often hold up in the false lumen after clearance of the true lumen. Type 3 mimics atherosclerosis with focal or tubular stenosis, often requiring intravenous ultrasound or optical coherence tomography to differentiate the cause [5]. If SCAD is suspected and coronary angiogram is unavailable or the clinical situation permits, noninvasive cardiac CTA is another available diagnostic modality. However, limitations include a low spatial resolution for small coronary vessels, motion artifact, and unknown sensitivity and specificity.

**Table 2:** Yip-Saw angiographic classification of spontaneous coronary artery dissection (SCAD).

	Yip-Saw angiographic classification of SCAD
Type 1	Two or more vessel lumens separated by a dissection flap
Type 2	Long, smooth, tapered narrowing of the vessel
Type 3	Focal stenosis of the vessel as seen in atherosclerosis

The goal of managing SCAD is to restore myocardial perfusion and preserve cardiac function, as with other forms of ACS. Adverse outcomes have been reported with the use of thrombolytics; therefore, thrombolysis is generally not indicated in the case of SCAD. Similarly, while PCI is the primary method of management in atherosclerotic ACS, it often results in poor outcomes in the management of SCAD, mainly hematoma propagation, iatrogenic dissection, and abrupt vessel occlusion. PCI is indicated, however, only in patients with ongoing ischemia or hemodynamic instability. A more conservative approach using similar guideline-directed medical management for ACS, such as beta-blockade, antiplatelet and statin therapy, and blood pressure control, is preferred in clinically stable patients without high-risk anatomy. Anticoagulation is not indicated for SCAD alone unless the patient has another comorbidity that would warrant therapy. The primary concern with anticoagulation would be the extension of the intramural hematoma, thus worsening the severity of the dissection. Coronary artery bypass grafting is reserved for cases refractory to conservative medical management with or without PCI.

The recurrence rate of SCAD ranges from 5% to 19% [5]. To reduce the rate of recurrence, management of risk factors is essential. It includes controlling blood pressure, diabetes, and cholesterol levels. Lifestyle modifications such as smoking cessation, limiting stress, and introducing mild-to-moderate aerobic exercise are indicated. Post-SCAD chest pain is common; therefore, antianginals such as nitrates, calcium channel blockers, or ranolazine may be warranted [6]. Based on clinical suspicion, periodic noninvasive monitoring with ECG, cardiac biomarkers, stress echo, and perfusion imaging may be necessary. Invasive coronary angiography is reserved for patients with more severe symptoms, along with concerning findings on noninvasive testing. Lastly, it is essential to have an emergency action plan for patients following SCAD so that they can seek appropriate care should symptoms recur.

## Conclusions

SCAD is a rare cause of ACS, with a greater prevalence in females that should be included in the differential in patients presenting with chest pain and equivalent symptoms. Noninvasive evaluation with ECG and cardiac biomarkers should be obtained initially; however, a definitive diagnosis usually requires coronary angiography. Most patients are treated with conservative medical management, with PCI and coronary artery bypass grafting reserved for unstable patients or those refractory to conservative management.
